# The geography of sex: sexual conflict, environmental gradients and local loss of sex in facultatively parthenogenetic animals

**DOI:** 10.1098/rstb.2017.0422

**Published:** 2018-08-27

**Authors:** Nathan W. Burke, Russell Bonduriansky

**Affiliations:** 1 School of Biological, Earth and Environmental Sciences, UNSW, Sydney, New South Wales, Australia; 2 Evolution and Ecology Research Centre, UNSW, Sydney, New South Wales, Australia

**Keywords:** geographical parthenogenesis, facultative parthenogenesis, paradox of sex, sexual conflict, environmental gradient, individual-based model

## Abstract

Obligately asexual organisms tend to occur at higher altitudes or latitudes and occupy larger ranges than their obligately sexual relatives—a phenomenon called geographical parthenogenesis. Some facultatively parthenogenetic organisms that reproduce both sexually and asexually also exhibit spatial variation in reproductive mode. Theory suggests that sexual conflict and mate limitation can determine the relative frequency of sex in facultative parthenogens, but the effect of these dynamics on spatial distributions is unknown. Here, we use individual-based models to investigate whether these dynamics can generate local differences in the reproductive mode in a facultatively parthenogenetic metapopulation occupying an environmental gradient. We find that selection for resistance and high fecundity creates positive epistasis in virgin females between a mutant allele for parthenogenesis and alleles for resistance, resulting in female-biased sex ratios and higher resistance and coercion towards the productive ‘core’ of the metapopulation. However, steeper environmental gradients, which lead to lower density and less mating at the ‘edge’, generate female bias without promoting coercion or resistance. Our analysis shows that local adaptation of facultatively parthenogenetic populations subject to sexual conflict and productivity gradients can generate striking spatial variation suggesting new patterns for empirical investigation. These findings could also help to explain the rarity of facultative parthenogenesis in animals.

This article is part of the theme issue ‘Linking local adaptation with the evolution of sex differences'.

## 1. Introduction

Sexual reproduction is paradoxical because it is associated with numerous costs that asexual organisms avoid [1,2]. The prevalence of sexual reproduction in complex organisms therefore requires explanation because parthenogenetic females do not pay such costs [3]. This means that, all else being equal, parthenogenesis should outcompete and supplant sex, at least in the short term.

Despite this predicted advantage, the evolution of parthenogenetic forms within obligately sexual lineages rarely results in the complete extinction of sex. More frequently, sexual and asexual relatives coexist within the same range while occupying distinct geographical areas or ecological niches. For example, asexuals tend to have larger ranges or more marginal distributions at higher latitudes or altitudes than their sexual counterparts [4,5], and often occupy territories associated with glacial retreat [5,6] or high disturbance [7,8]. This general pattern—coined geographical parthenogenesis [4]—is well documented in a diversity of taxa [9–12] and across various environments [11,13,14].

The ability of parthenogenetic females to produce offspring uniparentally is thought to be an important factor in geographical parthenogenesis because only one individual female or egg is required to establish a population at the range edge when reproduction is asexual [9,15,16]. Other potential mechanisms include outbreeding depression generated by asymmetrical gene flow from core (source) to marginal (sink) habitats [17], and lower capacity of dispersal in sexuals than asexuals [18]. A number of verbal models propose that correlates of parthenogenesis—such as polyploidy and hybridity—rather than parthenogenesis *per se* could provide advantages that drive geographical differences in reproductive mode [5,9]. However, other models suggest that asexuals might be more prevalent in marginal habitats because factors that constrain asexual success due to the narrower niche breadth of genetically invariable parthenogens operate to a lesser degree at population edges. For example, biotic interactions—such as parasitism, predation and competition—might be less intense, and resources may be less diverse or in shorter supply in marginal compared with core habitats [19–23].

An important assumption of current theory on geographical parthenogenesis is that sexual and asexual organisms are reproductively isolated ‘species’. Although this assumption is realistic for many sexual–asexual relatives (e.g. [24–26]), genetic isolation between sexual and asexual forms often varies between taxa [27,28], and such variation could influence spatial distributions. Facultative parthenogenesis is a reproductive strategy where sexual and asexual reproduction are not isolated in distinct ‘species’ but can occur in any individual female depending on whether mating takes place. Like obligately sexual and asexual sister taxa, some facultatively parthenogenetic organisms exhibit spatial variation in sex ratio along environmental gradients. For example, in the common tea-tree stick insect, *Clitarchus hookeri*, from New Zealand, equal sex ratios are found on the west coast of the North Island but sex ratios become increasingly female-biased towards the east, culminating in all-female populations on the South Island where the species' range appears to be expanding [29]. Interestingly, when South Island females are crossed with North Island males, far fewer sons are produced [29], suggesting an association between high rates of parthenogenesis and increased fertilization failure or resistance. Intriguingly, in two species of facultatively parthenogenetic Japanese harvestmen, *Leiobunum manubriatum* and *Leiobunum globosum*, males decline in number with increasing latitude and altitude [30], but males from the most female-biased populations exhibit exaggerated secondary sexual traits that are used for mate clasping and copulation [30]. In Japanese harvestmen, higher rates of parthenogenesis may therefore be associated with increased male coercion and sexual conflict. Beyond these suggestive examples, very little is known about spatial variation in metapopulations of facultative animals. Thus, it remains unclear whether the incidence of asexual reproduction in facultative taxa varies with environmental productivity in a similar way to geographical parthenogenesis in obligately sexual–asexual sister taxa. It is also not known what factors might generate geographical variation in facultative systems.

Uniparentality and mate limitation have been shown to feedback on each other to promote high rates of parthenogenesis in facultatively parthenogenetic *Timema* stick insects [31], and low dispersal between contiguous populations has been shown to drive female-biased sex ratios in *Drosophila mercatorum* [32]. However, other factors are also likely to be important. Many facultatively parthenogenetic animals exhibit lower fecundity via asexual reproduction than via sex [33,34], and such a constraint could affect the evolution of spatial variation. But fecundity differences have rarely been considered in the context of geographical parthenogenesis, because models have mostly assumed a twofold cost of sex (i.e. no constraints on parthenogenesis [35]). Recent work also suggests that sexual conflict over mating frequency could be particularly intense in facultatively parthenogenetic taxa because of the potential for female reproduction without mating [36]. A recent model suggests that female-biased sex ratios and higher rates of parthenogenetic reproduction are most likely when population density and costs of resistance are low [37]. Another theoretical study found that extinction of males is most likely if linkage disequilibrium can build up between parthenogenesis and resistance, thereby giving females the upper hand in sexual conflicts, whereas males can be maintained at low frequencies if coercion can counter-evolve [38]. Despite this work, it is currently unclear what roles sexual conflict and sexually antagonistic coevolution play in shaping geographical patterns in facultatively parthenogenetic systems because theoretical studies have so far only modelled single homogeneous populations (e.g. [37–40]).

Sexual conflict could play a role in geographical parthenogenesis in several ways. Alleles for facultative parthenogenesis might initially take hold at the uninhabited edge of sexual metapopulations because of the colonization advantage of uniparental reproduction when density is low (as outlined in [16] and [31]). Whether female-biased sex ratios persist at the range edge might then depend on the level of core-to-edge dispersal and the capacity of females to resist mating attempts. Sexual conflict over mating frequency could promote parthenogenesis at the core where density is highest, especially if alleles for parthenogenesis become linked with alleles for resistance. Furthermore, the demographic advantage of asexual reproduction (i.e. rapid population growth) could turn former sink populations into sources if fecundity via parthenogenesis is high, allowing parthenogens to rapidly swamp neighbouring populations containing males. However, such an effect may depend on spatial variation in environmental productivity. Local co-adaptation to sexual conflict could also contribute to the evolution of spatial differences in antagonistic traits. For example, if selection generates large numbers of females with higher than average resistance (as per [38]), males may need to counter-evolve higher than average coercion to persist in female-dominated populations.

We test these predictions using an individual-based model that simulates the invasion of a sexual metapopulation by facultatively asexual mutants across a range of ecological and genetic conditions. We investigate how sexual conflict interacts with other factors associated with geographical parthenogenesis—including colonization via uniparentality, ecoclines in productivity and probability of dispersal—to drive spatial differences in facultatively parthenogenetic populations. Specifically, we ask what conditions and dynamics lead to the evolution of female-biased sex ratios that result from higher rates of facultative parthenogenesis at either the range core or edge, and whether female bias is associated with higher levels of resistance and/or coercion.

## 2. Model

### (a) Overview

**Figure 1.**
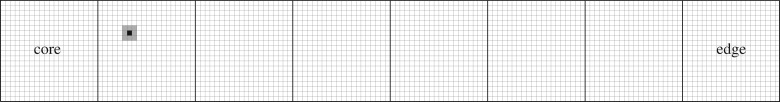
Spatial structure of the simulated environment, showing the eight linear habitats that decline in productivity from core to edge, and the *n* × *n* patches within each habitat. A female (back square) is shown surrounded by eight potential mates (grey squares).

We consider a metapopulation of obligately sexual diploid organisms with discrete generations undergoing sexually antagonistic coevolution while generating mutants capable of facultative parthenogenesis. We model facultative parthenogenesis in an invasion scenario because invasion following deglaciation is a common feature of geographical parthenogenesis [5], and spatial outcomes could depend on coevolutionary dynamics between reproductive mode and sexually antagonistic traits at the time of invasion. The metapopulation comprises eight distinct habitats (*n* × *n* patches each, where *n* = 20 and each patch possesses no more than one individual) arranged in a row with explicit boundaries to allow edge effects (figure 1). This structure is typical of metapopulation models involving dispersal and range expansion (e.g. [41–43]). To incorporate spatial variation in productivity (e.g. [44,45]), we assume a one-dimensional ecocline reflecting a linear decrease in environmental productivity (and therefore mean fecundity) from the left-most habitat (core) to the right-most habitat (edge).

Individuals possess a total of 21 biallelic, diploid, autosomal loci which describe coercion, resistance and reproductive mode. Sexual inheritance of these traits is Mendelian, whereas parthenogens inherit two random copies of their mother's alleles for each locus, allowing some recombination, as occurs in automixis [46]. We model coercion and resistance as polygenic traits each controlled by 10 loci with additive, sex-limited, co-dominant effects. The phenotypic values of male coercion and female resistance are, respectively:
2.1*a*
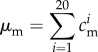
and
2.1*b*
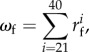
where 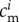 is the *i*th coercion allele carried by a male (loci 1–10, each with two alleles), and 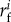 is the *i*th resistance allele carried by a female (loci 11–20, each with two alleles). The relative values of these phenotypes determine whether mating takes place (see *Mating*, below). Our use of a polygenetic architecture is motivated by the observation that coercion and resistance phenotypes frequently comprise numerous morphological, physiological and behavioural characteristics probably controlled by multiple loci [47,48].

Mutation at a single locus is one of many possible routes to asexuality in animals [49]. Therefore, for simplicity, we assume that a single, additional locus (locus 21) with two alleles (*p* and *P*) controls the reproductive mode, where *pp* is the wild-type sexual genotype, and *P* is a dominant mutant allele with a female-limited effect that allows virgin females to produce daughters asexually from unfertilized eggs (i.e. via facultative parthenogenesis). To minimize the influence of drift on the fate of the *P* allele, we assume that the *P* allele arises in a single mutation event in a random quarter of individuals from the core population, with half the mutants becoming *pP* and half becoming *PP*. This very high mutation rate is unlikely to bias our results, because qualitatively similar outcomes are obtained with a mutation rate of 1%, and when all de novo mutants are heterozygotes (*pP*) (see electronic supplementary material, figure S5).

Our model assumes sexual conflict over mating frequency such that females lose fitness if they mate more than once. Fitness (lifetime number of offspring) is modelled as a function of the number of matings a female achieves, modified by a cost of resistance and a penalty for reproducing outside the core environment. For obligately sexual and mutant females, fitness is calculated as follows:
2.2*a*
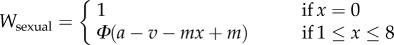
and 
2.2*b*
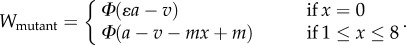


Here, the number of matings, *x*, is limited to a maximum of 8, as this is the largest number of male neighbours available to females as mates (figure 1 and see *Mating*, below). *m* is the rate of change in fecundity as *x* increases following the first mating, where increasing values of *m* generate more intense sexual conflict (as per [50,51]). *a* specifies the fecundity maximum when *x* = 1. *ɛ*, which is bound between 0 and 1, is a multiplier that determines the fecundity of parthenogenetic reproduction relative to *a*. *v* = *dκ* is the penalty to fecundity driven by the ecocline, where *d* is the distance in the number of habitats from the core (where 0 ≤ *d* ≤ 7) and *κ* is a constant controlling the steepness of the decline. *Φ* = 1 − *ζω*_f_*h* is the cost of resistance (bound between 0 and 1), where *ω*_f_ (from equation (2.1*b*)) is the number of resistance alleles carried by a female, *h* is the number of mating attempts a female experiences (i.e. number of neighbouring males) and *ζ* is a constant controlling the steepness of the decline. Examples of these fitness functions are shown in electronic supplementary material, figure S1. Differences between the first pieces of equations (2.2*a*) and (2.2*b*) reflect the ability of mutants to obtain non-zero fitness as virgins [36] as well as physiological and reproductive costs associated with parthenogenesis [33,34,52]. Virgin mutants have equivalent fecundity to once-mated females (mutant or wild-type) when *ɛ*= 1, and lower fecundity when *ɛ* < 1, such that mating once is never costly for any female. Fecundities are otherwise equivalent between mutant and wild-type females for values of *x* > 0 (as evidenced by the identical second pieces in equations (2.2*a*) and (2.2*b*)).

## 3. Initialization and burn-in

Simulations were initialized with *N* = *n*^2^ adults per habitat (where *N* = 400 = local carrying capacity). All initialized individuals possessed the wild-type *pp* genotype for the reproductive mode and a randomly assigned sex. The two alleles at each of the 10 resistance loci and 10 coercion loci were initialized by twice drawing a random number from the discrete uniform distribution *U*{0, 1}. The *P* allele was introduced at generation 50 when frequencies of antagonistic alleles had dropped sharply and had begun to approach equilibrium (see electronic supplementary material, figure S6). We wanted to ensure the existence of standing genetic variation in coercion and resistance at the point of *P* allele introduction because the evolution of resistance has been suggested as a key factor in the spread of facultative parthenogenesis [36,37,39], and we were interested in its influence on patterns of geographical parthenogenesis.

Given that many examples of geographical parthenogenesis are associated with colonization of new habitats from glacial refugia [5,6,53], we assessed the influence of refugia by initializing simulations with varying metapopulation sizes such that (i) all habitats were initially habitable (no refuge), (ii) only the four left-most habitats of the metapopulation were initially habitable (large refuge) or (iii) only the core habitat was initially habitable (small refuge). Uninhabitable areas were switched to habitable upon the introduction of the *P* allele at the end of the 50-generation burn-in period, simulating the opening of new habitats following glacial retreat. The simultaneous scheduling of these two events—deglaciation and origin of mutations for parthenogenesis—was motivated by the apparent link between rapid climate change and parthenogenesis in natural populations [35,54].

## 4. Life cycle

**Figure 2.**
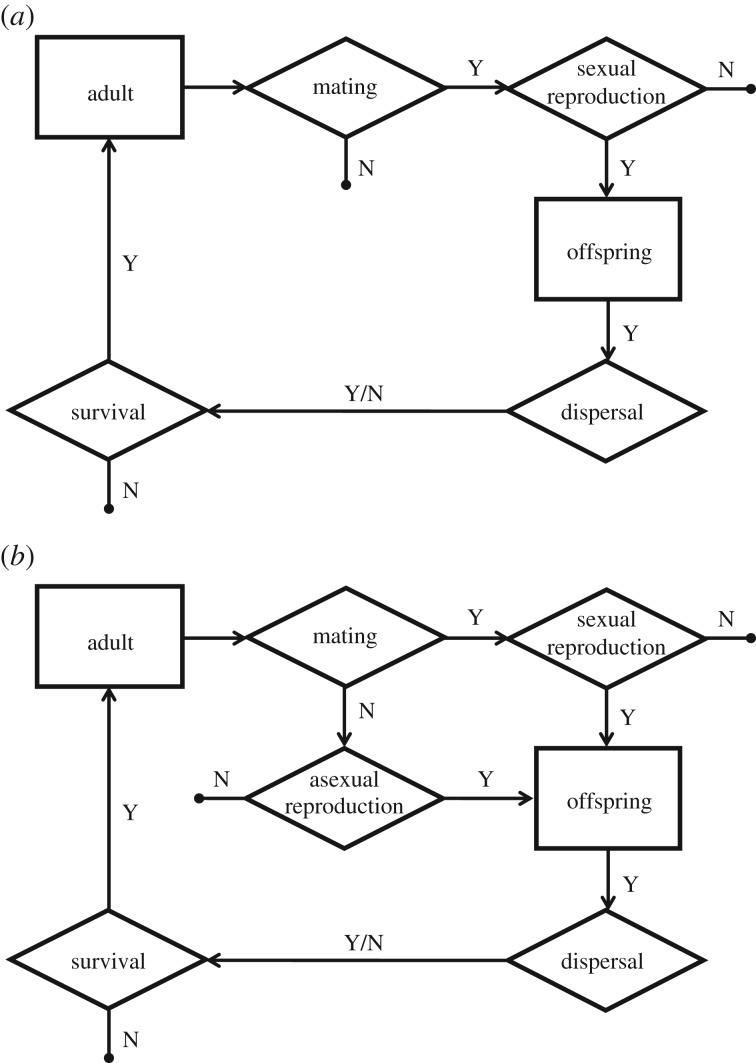
Life cycle of sexual (*a*) and mutant (*b*) organisms, showing the progression of life stages (squares) and the processes that occur at each stage (diamonds).

During each generation, individuals perform tasks in the following temporal order (see figure 2 for a schematic of the life cycle).

### (a) Mating

Males attempt to mate with females from the same habitat that shares an edge or vertex with their own patch (i.e. ‘neighbouring females'). The number of potential mates, *h*, per individual is therefore ≤8 (figure 1). Both obligately sexual and mutant females can mate and reproduce sexually. Mating is attempted if a male's coercion value is greater than or equal to a neighbouring female's resistance value (i.e. if *μ*_m_ ≥ *ω*_f_). However, we assume that the likelihood of successful mating with any female (irrespective of her level of resistance) decreases linearly with increasing male coerciveness and mating number according to the function *J* = 1 − *ξμ*_m_*q*, where *J* is bound between 0 and 1, *μ*_m_ (from equation (2.1*a*)) is the total number of coercion alleles carried by a male, *q* is the number of matings a male has already achieved and *ξ* is a constant controlling the steepness of the decline. Thus, mating attempts are successful if *J* is greater than a randomly selected number between 0 and 1. This function represents a trade-off with coerciveness. Although the costs of expressing coercive secondary sexual traits are poorly understood [55], trade-offs are a biologically plausible way to model the costs of coercion [56,57]. For example, a male bearing a more effective coercive trait (e.g. larger clasping appendages) may have a higher probability of mating with a highly resistant female, but may also be less agile and therefore have reduced probability of mating with other females [58]. We assume that mated females store enough sperm from one mating to fertilize all their eggs. We also assume that mutant females that receive sperm forgo the possibility of reproducing via parthenogenesis, which is typical of facultatively parthenogenetic taxa that store sperm [59].

### (b) Reproduction and inheritance

Obligately sexual and mutant females produce *W* offspring, rounded to the nearest integer. Mated females randomly choose the sperm of one of their previous mating partners to fertilize all their eggs, which results in sons and daughters with equal likelihood. Unmated mutants produce daughters only. Sexual inheritance follows Mendelian rules of segregation (i.e. one randomly chosen allele for each trait from each parent). Asexually produced offspring inherit two randomly selected copies of their mother's alleles for each locus. We assume no linkage between loci. Adults die simultaneously following reproduction.

### (c) Dispersal between habitats

Dispersal between habitats occurs at the offspring stage. Individual offspring that draw a randomly selected number between 0 and 1 that is less than the dispersal probability, *γ*, disperse to a randomly selected neighbouring habitat (i.e. a habitat that shares a boundary edge with the dispersers' home habitat)*.* Because dispersal is a fixed *per capita* probability, the number of dispersers increases with increasing offspring population size.

### (d) Survival and recruitment

We assume that the number of recruits per habitat is constrained by the local carrying capacity, *N*. If the number of offspring in a habitat (denoted by *ρ*_habitat_) exceeds *N*, a total of *ρ*_habitat_–*N* offspring are randomly selected to die. Surviving offspring then settle randomly on a vacant patch within their current habitat and simultaneously mature into adults. The life cycle repeats thereafter.

## 5. Simulation experiments

Simulations were run for 500 generations in the individual-based modelling program NetLogo [60] using custom-written code (available in the electronic supplementary material). Fifty independent runs were performed for each unique parameter combination. Parameter settings for the main model are shown in electronic supplementary material, table S1. To assess patterns of geographical parthenogenesis, we recorded sex ratios and the frequencies of coercion and resistance alleles at the end of simulation runs. We interpreted higher female bias as evidence of higher incidence of parthenogenesis.

### (a) Robustness analysis

To test the robustness of simulation outcomes to the underlying assumptions of the model, we reran a subset of simulations with sexual coevolution, costs of resistance and costs of coercion independently removed, and with maximum productivity, *a*, independently increased or decreased. Each perturbation was run 50 times. We used Vargha–Delaney effect-size *A*-tests from the effsize R package [61] to calculate the proportion of perturbed simulations that resulted in higher responses than baseline settings. Qualitatively large effect sizes (i.e. *A* ≥ 0.8 or *A* ≤ 0.2) indicated outcomes that were not robust to changes in underlying assumptions. A subset of the main model parameter space that generated spatial differences across all ecoclines and all response variables was chosen as the baseline for this analysis (see electronic supplementary material, table S1). Robustness results are provided in electronic supplementary material, table S2.

### (b) Sensitivity analysis

To test the sensitivity of simulation outcomes to fine-scale perturbations in the numerical parameter values used in simulation experiments, we ran a global sensitivity analysis using the Latin-hypercube sampling technique from the spartan R package [62]. This technique divides the range of each parameter of interest into a defined number of bins and combines them to create a hypercubic space of all possible parameter combinations. Continuous values of each parameter are then randomly selected from bins chosen at random from the hypercube without replacement, generating unique sets of test parameters for simulation runs. For our analysis, we generated 100 sets of test parameters from a hypercube constructed from 100 bins each of *κ*, *m*, *ɛ* and *γ.* Parameter ranges used to generate the hypercube are shown in the electronic supplementary material, table S1. We ran 50 simulations on each set and calculated Spearman's partial rank correlations between median response variables and predictor variables. Significant correlations indicated parameters that covaried with response variables irrespective of changes in other parameters. Sensitivity results are provided in electronic supplementary material, table S3.

## 6. Results

### (a) Invasion by facultative parthenogenesis

High fecundity of parthenogenetic reproduction relative to sexual reproduction (0.7 ≤ *ɛ* ≤ 1) results in rapid and complete displacement of alleles for obligate sex by the invading *P* allele, generating populations that are strictly facultatively parthenogenetic (i.e. with all females having the capacity to reproduce either sexually or asexually). Lower parthenogenetic fecundity (*ɛ* < 0.7) results in either the coexistence of both wild-type and mutant alleles or much slower invasion by the *P* allele, but generates no spatial variation in sex ratio in either case (results not shown). Values of *ɛ* > 1 result in all-female metapopulations (results not shown). Because we are primarily interested in understanding conditions that generate spatial patterns in facultative parthenogens, we focus here on results for 0.7 ≤ *ɛ* ≤ 1.

### (b) What conditions generate spatial variation in sex ratio?

The efficiency of uniparental reproduction allows mutants originating from small and large refugia to readily invade newly available habitats towards the edge following simulated glacial retreat. However, this advantage is quickly lost as immigrant males disperse and produce sons, evening out the sex ratio towards the edge. This can be seen in the sharp spike and rapid decline in sex ratio at the edge near generation 100 in electronic supplementary material, figure S2. This initial colonization effect is consistent across parameter settings, but other factors determine what happens to sex ratios thereafter.

**Figure 3.**
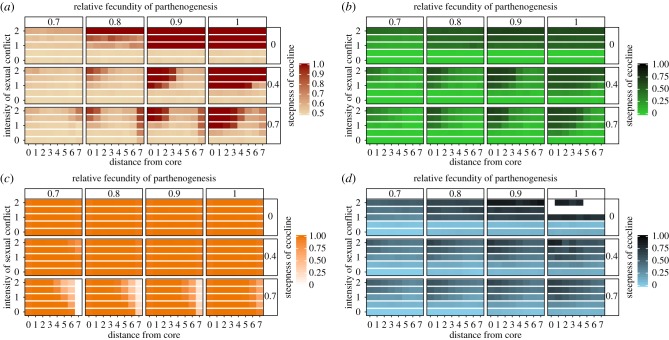
Heat maps showing spatial patterns for sex ratio (*a*), frequency of pooled resistance alleles (*b*), population density (*c*) and frequency of pooled coercion alleles (*d*) in metapopulations with no initial refugia. The core population is represented by distance 0 on the bottom *x*-axis; distance 7 is the edge population. The top *x*-axis shows the relative fecundity of parthenogenesis, *ɛ*. The left-hand *y*-axis depicts *m*, the female fitness gradient, which controls the intensity of sexual conflict. The right-hand *y*-axis shows the steepness of the ecocline, *κ*. Values closer to 1 signify more female-biased sex ratios (*a*), higher frequencies of resistance alleles (*b*), higher densities (*c*), and higher frequencies of coercion alleles (*d*). White regions in panel (*d*) indicate male extinctions. Outcomes are median proportions obtained from 50 simulation runs lasting 500 generations each. (Online version in colour.)

**Figure 4.**
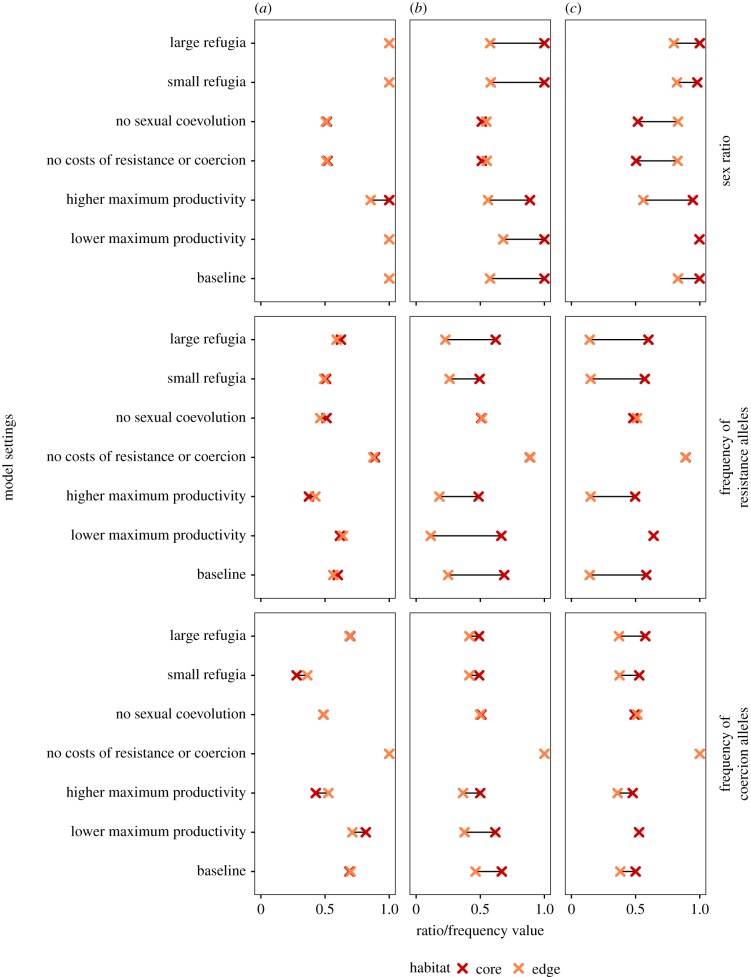
Segment graphs showing core-to-edge differences in sex ratio, frequency of pooled resistance alleles and frequency of pooled coercion alleles following relaxation of model assumptions from baseline settings for simulations with no global fecundity ecocline (*κ* = 0) (*a*), a shallow ecocline (*κ* = 0.4) (*b*) and a steep ecocline (*κ* = 0.7) (*c*). Crosses denote median ratios/frequencies for core and edge populations obtained from 50 simulation runs. Segment lengths indicate the size of core-to-edge differences. Parameters controlling sexual coevolution, costs of coercion and resistance, level of maximum productivity and refugia size were perturbed independently of each other. Baseline parameter settings are listed in electronic supplementary material, table S1. (Online version in colour.)

Mate limitation is the primary driver of sex bias at the edge. Steep productivity ecoclines (i.e. high values of *κ*) generate low density at the edge (figure 3*c*) and therefore high mating failure. Females that fail to mate reproduce asexually, generating female-biased sex ratios (figure 3*a*). However, while sex ratios remain female-biased at the edge, sex ratio also fluctuates rapidly and continuously (see electronic supplementary material, figure S2C) due to frequency-dependent selection: sex becomes more frequent as female numbers increase, but the resulting increase in males elevates the rate of costly mating, which in turn reduces female fecundity and density, and allows parthenogenetic reproduction to proliferate again (electronic supplementary material, figure S3). Because productivity is low at the edge, a further reduction in female fecundity as a result of increased mating rate causes density to decline well below the carrying capacity and selects for parthenogenesis (but not resistance) because many females fail to encounter males. Robustness analysis shows that elevated female bias at the edge is unaffected by the size of refugia, sexual coevolution, costs of coercion or costs of resistance (figure 4*c*; electronic supplementary material, table S2), because these factors have little influence on density. However, factors that raise density—such as lower costs of mating for females (i.e. less intense sexual conflict, *m*; figure 3*a*) and higher maximum per-female productivity (figure 4*c*)—reduce female bias at the edge by making sex less costly. Sensitivity analysis shows that dispersal probability has little influence on sex ratios at the edge or the core (electronic supplementary material, table S3).

**Figure 5.**
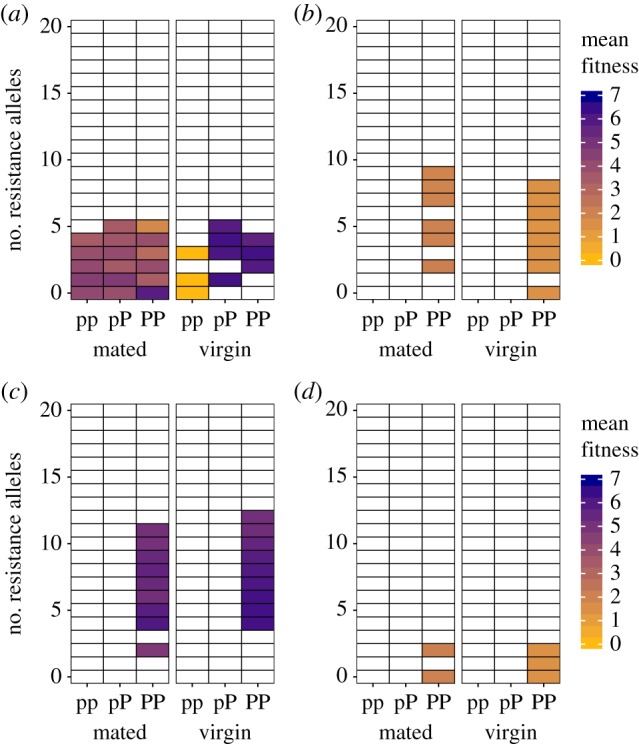
Heat maps showing mean female fitness as a function of resistance allele number (*y*-axis), reproductive-mode genotype (*x*-axis, top row) and mating status (*x*-axis, bottom row). Each plot is a snapshot of female fitness at the core (*a*,*c*) and the edge (*b*,*d*) at time-step 75 (*a*,*b*) and time-step 250 (*c*,*d*) from a single simulation run. During the early stages of invasion, positive epistasis for fitness between the *P* allele and resistance alleles occurs at the core (*a*), where *pP* and *PP* genotypes are associated with a larger number of resistance alleles than the *pp* genotype, and where these combinations of alleles for parthenogenesis and resistance achieve higher fitness when mating is avoided. By contrast, at the edge (*b*), there is no association between parthenogenesis or resistance genotype and fitness. Following fixation of the *P* allele, females at the core carry more resistance alleles (*c*) than females at the edge (*d*) as a consequence of this past epistasis. Other parameters: small refugia, *κ* = 0.7, *m* = 1.5, *ɛ* = 0.9. (Online version in colour.)

Despite the consistent effect of mate limitation at the edge, sexual conflict has a stronger influence on sex ratios overall, particularly towards the core (figures 3 and 4). Robustness analysis shows that core populations exhibit female-biased sex ratios regardless of the initial presence or size of refugia or the steepness of the fecundity ecocline (figure 4; electronic supplementary material, table S2). This is because high productivity and selection for resistance at the core generate positive epistasis for fitness between resistance alleles and the *P* allele, leading to the build-up of linkage disequilibrium between these traits (figure 5). Once the *P* allele fixes, this epistatic interaction results in more resistance alleles at the core than the edge (compare figure 5*c* and *d*). Resistance enhances opportunities for parthenogenesis at the core by enabling females to avoid costly mating, while high productivity promotes the spread of resistance alleles via asexually produced daughters. High productivity has this effect because of the (up to) twofold advantage of producing all-female offspring via parthenogenesis (i.e. the so-called twofold cost of sex [3]). This advantage explains why female bias at the core does not evolve at low values of *ɛ* (figure 3*a*): lower parthenogenetic output inhibits the rapid spread of resistance alleles, thereby generating weaker epistasis. The fact that sex bias fails to evolve at lower values of *ɛ* (figure 3*a*) even though core populations are equally dense across all values of *ɛ* (figure 3*c*) suggests that high productivity rather than high density *per se* favours epistasis at the core.

Female bias fails to evolve at the core when resistance alleles are neutral and therefore sexual coevolution does not occur (figure 4; electronic supplementary material, figure S4). Removing costs of resistance and costs of coercion has a similar effect (figure 4) because coercion alleles fix in the absence of costs and males subsequently succeed in all mating attempts. However, removing sexual coevolution or costs of coercion and resistance from the model does not reduce female bias at the edge: mate limitation still selects for higher rates of parthenogenesis when the ecocline is steep (figure 4*c*; electronic supplementary material, figure S4A). Removing these assumptions also has no effect on sex-ratio fluctuations when mating is costly at the edge (described above), which indicates that reduced fecundity from mating rather than sexual coevolution drives these fluctuations.

### (c) What conditions generate spatial differences in female resistance and male coercion?

Selection for high resistance and high productivity creates positive epistasis for fitness between the *P* allele and resistance alleles, leading to high resistance in sex-biased populations (compare spatial patterns of figure 3*a*,*b*). However, low resistance evolves in female-biased edge populations because low density driven by low productivity causes weak selection on antagonistic traits (figure 3*a*,*b*).

Male coercion and female resistance show correlated evolution, as expected under sexually antagonistic coevolution (compare figure 3*b* and *d*). Males regularly fail to mate at the core and sons are rarely produced there because of strong selection for female resistance and parthenogenesis: only males with high coercion values can persist at the highly resistant, female-dominated core. Hence, coercion is typically higher at the core than at the edge (figure 3*d*).

## 7. Discussion

We identified sexually antagonistic coevolution and mate limitation as the most important drivers of geographical parthenogenesis in facultative taxa. Spatial distributions were largely dependent on how these forces played out locally under varying levels of environmental productivity.

Our model predicts that low density caused by steep declines in environmental productivity will be the primary driver of parthenogenesis at the range edge because mating is either too difficult or too costly in such environments. This prediction is in agreement with previous suggestions that female-only populations will be more likely at range peripheries where mates may be difficult to find or males absent by chance [63,64]. Such dynamics are thought to underpin distributions of reproductive mode in at least some facultatively asexual animals (e.g. [31,32]). Our model extends this mate-limitation hypothesis by showing that, in addition, costs of mating can drive negative frequency-dependent selection for parthenogenesis in low-productivity habitats, potentially resulting in cyclically fluctuating frequencies of parthenogenetic reproduction. Importantly, our model also demonstrates that, in the absence of resistance, environmental gradients can generate female bias at the edge but not at the core, suggesting that constraints on the ability of females to evolve effective resistance could explain spatial patterns in natural populations. We also show that lower levels of resistance and coercion are expected in edge environments because low density generates relatively weak selection on these traits by comparison with the core habitat.

Our model predicts that sexual conflict will promote higher rates of parthenogenesis at the range core where selection for female resistance is strongest. High female fecundity via parthenogenesis allows resistance alleles to spread rapidly at the core, generating linkage disequilibrium and strong epistasis for fitness when mating is avoided. These dynamics lead to widespread sex-ratio bias and heightened resistance across the most productive part of the range. But populations of slightly lower productivity are less likely to be swamped by highly resistant parthenogens and so maintain unbiased sex ratios and weaker epistasis. Selection for resistance in the absence of local variation in productivity, however, results in the widespread extinction of males.

These findings contrast with some results obtained in previous theoretical studies. In a model of haploid, facultatively parthenogenetic organisms in which only resistance could evolve, Gerber & Kokko [37] found that intense conflict under high densities favoured sex because accepting multiple matings was less costly for females than resisting them. Our findings suggest an alternative pattern: in our model, parthenogenesis proliferated in highly productive, densely populated habitats—even when resistance was costly—because larger numbers of coercive males generated stronger selection on females to resist, and high productivity allowed resistance and parthenogenesis to spread together. These differences may reflect the fact that we modelled sexual conflict as a reduction in female fitness as mating number increased over 1, whereas Gerber & Kokko's model assumed no costs of mating, only costs of resistance [37]. Thus, the nature of sexual antagonism could be important in determining outcomes of alternative reproductive modes.

The extent to which sexual conflict drives female bias in facultatively parthenogenetic populations is currently unclear because of a lack of data on sexual conflict in such systems. Nevertheless, our model suggests three preconditions that are jointly sufficient for sexual conflict to generate and maintain geographical patterns of reproductive mode in facultative taxa: (i) females must experience sexual conflict over mating frequency to drive evolution of resistance; (ii) the fecundity of females reproducing via parthenogenesis must be high enough to allow resistance alleles to spread rapidly; and (iii) there must be spatial variation in environmental productivity so that males do not die out in all parts of the range. If these preconditions are met, our model predicts certain outcomes. First, linkage disequilibrium between resistance and parthenogenesis should result in female-biased populations with higher resistance than unbiased populations. Second, rare males in female-biased populations should exhibit exaggerated coercive traits compared with males from unbiased populations. While there is some anecdotal support for these predictions [29,30,65–67], more empirical data are needed. For example, large parts of the southern range of the New Zealand common tea-tree stick insect have no males, and females from these populations perform poorly when crossed with males from northern regions [29], suggesting higher resistance to fertilization in more female-biased habitats. However, low fertilization success could also reflect ongoing reproductive isolation between geographically distant populations. A positive correlation between coercion, resistance and female-biased sex ratio would support the sexual conflict hypothesis.

One of our key findings is that intense sexual conflict can, under certain circumstances, lead to obligate parthenogenesis (via male extinction) across the entire range (figure 3*a*). When this occurs in nature, signatures of high resistance should be observable in obligately parthenogenetic taxa but not in their sexual relatives. Evidence from *Timema* stick insects is consistent with this prediction: strong rejection behaviours occur in obligately asexual populations that share no range overlap with sexual relatives, whereas females in obligately sexual populations rarely reject copulation attempts [68]. These resistance behaviours make little sense in the context of present-day selective pressures but could be evidence of past selection for resistance that led to the extinction of males in this particular lineage. Past selection for resistance could also explain the rapid loss of female sexual traits associated with mate attraction, sperm storage and fertilization in obligately asexual taxa [69,70].

Interestingly, we found no long-term association between post-glacial range expansion and spatial patterns. This was surprising as such a link is inferred in numerous asexual species and their sexual progenitors [5,35], and in some facultatively parthenogenetic taxa [29,67]. In our model, parthenogens rapidly colonized recently deglaciated regions via the advantage of uniparentality, but female-biased sex ratios quickly equalized as dispersing males arrived and mated with resident females. The sex ratio was thereafter determined by mate availability, sexual conflict and local productivity rather than historical range expansion. Yet in nature, female-biased populations from deglaciated habitats appear to be immune to male invasion (e.g. [67]), despite the large reproductive advantage to immigrant males as the rarer sex [71]. What maintains widespread female bias in deglaciated regions? Our model suggests three possible explanations. First, the colonization phase of deglaciation could still be ongoing, and males may be absent because they have not yet arrived. This is unlikely given the timescales involved, but observed cases of ongoing range expansion (e.g. [29]) lend credence to this possibility. Second, environmental gradients in natural populations may be nonlinear such that low densities cover much more of the post-glacial region than just the extreme edge, leading to widespread chronic mate limitation and female bias. Third, resistant females could exclude males from post-glacial regions by avoiding mating. Our model predicted that sexual conflict should select for parthenogenesis and female bias whenever mating is costly and productivity is locally high. Whether in natural populations female-biased populations have higher productivity than other populations in the range remains unexplored. Such data may help to clarify whether higher coercion at female-biased edge populations of two Japanese harvestmen [30] is driven by high productivity as predicted by our model.

High rates of dispersal can swamp locally adapted genotypes and dissolve spatial differences (as per [72,73]), but we found little effect of dispersal on core-to-edge variation. This was because sexual coevolution allowed females at the core to adapt to influxes of coercive males by evolving higher resistance. However, we also assumed that dispersal was sex-independent and occurred only between neighbouring habitats, whereas many animals exhibit sex-biased dispersal [74,75] and some can disperse long distances [76]. In our simulations, higher rates of dispersal by females would probably enhance the spread of parthenogenesis throughout the metapopulation, leading to increased male extinction. The consequences of greater dispersal by males may depend on the dynamics of sexually antagonistic coevolution. For example, higher male dispersal might reduce the incidence of parthenogenetic reproduction if female resistance is limited or resistance is very costly. However, because increased male dispersal would generate stronger selection for female resistance, it is unlikely to eliminate spatial variation in sex ratio and reproductive mode.

We assumed that the mutant allele for facultative parthenogenesis arose in a large proportion of the core population and at a single point in time in order to limit the effects of genetic drift. However, although such a high rate of mutation is biologically improbable, the initial frequency of the allele did not appear to affect evolutionary outcomes as very low and very high initial frequencies of *P* generated comparable results (compare figure 3 and electronic supplementary material, figure S5). The simulation burn-in period allowed sexual coevolution to stabilize while still preserving some genetic diversity in coercion and resistance traits. High genetic diversity may be characteristic of sexually antagonistic traits, given the multi-trait nature of resistance and coercion phenotypes [48] and the large number of sexually antagonistic loci throughout the genome [47]. Assuming a high level of genetic variation for these traits allowed us to observe sexual coevolution in response to the introduction of the *P* allele and to clearly determine the contribution of resistance to spatial distributions of parthenogenesis. In itself, this polygenic architecture probably had little effect on selection for parthenogenesis in our model, but larger effects could occur if resistance loci reside on multiple chromosomes. We also assumed that resistance and coercion traded off against reproductive success in similar ways for each sex. Assuming a different cost structure might alter expected outcomes because larger costs of resistance might constrain the evolution of high resistance genotypes and therefore limit rates of parthenogenesis.

Previous work suggests that linkage disequilibrium between resistance alleles and alleles for parthenogenesis, generated by epistasis for fitness between resistance and the capacity for parthenogenetic reproduction, could play an important role in the establishment of parthenogenetic populations [38]. Results of the simulations reported here show that positive epistasis for fitness between resistance and parthenogenesis can also generate spatial variation in sex ratio. However, linkage disequilibrium between these traits may be unlikely to arise in natural populations due to strong selection against extreme resistance in females [36,38]. Indeed, the low likelihood of parthenogenesis alleles arising within a genetic background of high resistance has been suggested as a potential explanation for the rarity of facultative parthenogenesis in nature [36]. Such a genetic constraint might also explain why female bias occurs only at the range edge in some taxa (i.e. due to mate limitation alone). Future work could provide valuable insights on the role of sexual conflict in facultative systems by documenting the extent of linkage disequilibrium in facultative populations.

Facultatively parthenogenetic animals offer valuable opportunities to understand the factors that contribute to the maintenance of loss of sexual reproduction, and may therefore hold clues to resolving the paradox of obligate sex [36]. But why facultative taxa exhibit geographical variation in sex ratios, female resistance and male coercion is an unresolved question. Our analysis suggests that variation in productivity along an environmental gradient drives local variation in patterns of sexual coevolution, resulting in spatial variation in reproductive mode and sex ratio. Our analysis also shows how sexual conflict and mate limitation can interact to generate geographical parthenogenesis in facultative taxa. More data from natural populations of facultatively parthenogenetic animals are needed to test our predictions.

## Supplementary Material

Supplementary material

## Supplementary Material

NetLogo code

## Supplementary Material

NetLogo code for low mutation rate simulations

## Data accessibility

Code used to run the simulations is available in the electronic supplementary material.
