# Correction to ‘The geography of sex: sexual conflict, environmental gradients and local loss of sex in facultatively parthenogenetic animals’

**DOI:** 10.1098/rstb.2019.0001

**Published:** 2019-02-11

**Authors:** Nathan W. Burke, Russell Bonduriansky

*Phil. Trans. R. Soc. B* **373**, 20170422. (Published 27 August 2018). (doi:10.1098/rstb.2017.0422)

There was an error in equation (2.2*a*) in our published paper; ‘*W*_sexual_ = 1’ should have read ‘*W*_sexual_ = 0’. The correct equation is as follows:

2.2a $$W_{\mathrm{sexual}}=\{\begin{array}{cc} 0\;\; & \mathrm{if}\;x=0\;\;\\ \Phi (a-v-mx+m) & \mathrm{if}\;1\leq x\leq 8 \end{array}$$

