# Supplementary material for: The geography of sex: sexual conflict, environmental gradients and local loss of sex in facultatively parthenogenetic animals
Source: Philos Trans R Soc Lond B Biol Sci. 2018 Aug 27;373(1757):20170422. doi: 10.1098/rstb.2017.0422 (PMC6125730; doi:10.1098/rstb.2017.0422)
Supplement: Supplementary material [file rstb20170422supp1.docx]

# **TABLE S1**

Model parameters, settings and descriptions.

| Parameter |  | Description |  | Settings for  main sim.  runs |  | Settings for  robustness testing |  | Settings for sensitivity  testing |
| --- | --- | --- | --- | --- | --- | --- | --- | --- |
|  |  |  |  |  |  |  |  |  |
| $refugia$ |  | Initial home range of the metapopulation |  | none |  | small ^*^  large ^*^  none_1_ |  | small |
|  |  |  |  |  |  |  |  |  |
| $m$ |  | Rate of change in the production of sexual offspring  as a function of mating number |  | 2  1.5  1  0.5  0 |  | 1.5_1_ |  | (0, 2) |
|  |  |  |  |  |  |  |  |  |
| $\varepsilon$ |  | Relative fecundity of parthenogenesis relative to $a$ |  | 0.7  0.8  0.9  1 |  | 0.9_1_ |  | (0.4, 1) |
|  |  |  |  |  |  |  |  |  |
| $\gamma$ |  | Probability of dispersal |  | 0.05 |  | 0.05_1_ |  | (0.005, 0.5) |
|  |  |  |  |  |  |  |  |  |
|  |  | Steepness of the global ecocline in fecundity from core to edge |  | 0  0.4  0.7 |  | 0_1_  0.4_1_  0.7_1_ |  | (0, 0.7) |
|  |  |  |  |  |  |  |  |  |
| $a$ |  | Maximum per-female productivity |  | 7 |  | 5 ^*^  7_1_  9 ^*^ |  | 7 |
|  |  |  |  |  |  |  |  |  |
| $\xi$ |  | Steepness of the cost of coercion |  | 0.01 |  | 0 ^*^  0.01 |  | 0.01 |
|  |  |  |  |  |  |  |  |  |
| $\zeta$ |  | Steepness of the cost of resistance |  | 0.01 |  | 0 ^*^  0.01_1_ |  | 0.01 |
|  |  |  |  |  |  |  |  |  |
| $sexual$  *coevolution* |  | The capacity of females to resist and drive sexual arms races |  | on |  | on_1_  off ^*^ |  | on |
|  |  |  |  |  |  |  |  |  |

Robustness settings with asterisks and subscript *1* denote perturbed and baseline values, respectively.

Brackets show the range of values (min, max) used in Latin-hypercube sampling for sensitivity tests

Small = core habitat; Large = left-hand half of metapopulation; None = all habitats

On = Resistance alleles functional; Off = Resistance alleles neutral

# **TABLE S2**

Vargha-Delaney *A*-test effect sizes for robustness analyses. Effect sizes represent the proportion of 50 simulation runs in which independent perturbations resulted in higher responses than baseline settings.

|  |  | No ecocline | | | Shallow ecocline | | | Steep ecocline | | | |
| --- | --- | --- | --- | --- | --- | --- | --- | --- | --- | --- | --- |
| Parameter perturbation | Habitat | Sex  Ratio | Freq. of resistance alleles | Freq. of coercion alleles | Sex  Ratio | Freq. of resistance alleles | Freq. of coercion alleles | Sex  Ratio | Freq. of resistance alleles | Freq. of coercion alleles |  |
| *sexual coevolution* = off | core | **0.01** | 0.33 | **0.16** | **0.01** | **0.15** | 0.28 | **0.01** | 0.31 | 0.44 |  |
|  | edge | **0.01** | 0.32 | **0.10** | 0.30 | **0.98** | 0.65 | 0.50 | **1.00** | **0.92** |  |
| $\zeta$ and$\xi$ = 0 | core | **0.01** | **0.99** | **1.00** | **0.01** | **1.00** | **1.00** | **0.01** | **1.00** | **1.00** |  |
|  | edge | **0.01** | **0.99** | **1.00** | 0.35 | **1.00** | **1.00** | 0.52 | **1.00** | **1.00** |  |
| $a$ = 9 | core | 0.67 | 0.79 | **0.82** | 0.66 | 0.77 | 0.71 | 0.60 | 0.59 | 0.51 |  |
|  | edge | 0.68 | 0.73 | **0.81** | 0.52 | 0.58 | 0.76 | **1.00** | 0.49 | 0.62 |  |
| $a$ = 5 | core | 0.57 | 0.58 | 0.67 | 0.50 | 0.41 | 0.48 | 0.45 | 0.53 | 0.50 |  |
|  | edge | 0.58 | 0.64 | 0.48 | **0.95** | **0.15** | **0.18** | NA | NA | NA |  |
| *refugia* = small | core | 0.67 | 0.68 | 0.78 | 0.61 | 0.79 | 0.74 | 0.59 | 0.54 | 0.49 |  |
|  | edge | 0.66 | 0.62 | 0.78 | 0.40 | 0.46 | 0.63 | 0.56 | 0.50 | 0.51 |  |
| *Refugia* = large | core | 0.48 | 0.56 | 0.49 | 0.48 | 0.33 | 0.32 | 0.51 | 0.57 | 0.61 |  |
|  | edge | 0.50 | 0.55 | 0.46 | 0.58 | 0.43 | 0.35 | 0.40 | 0.49 | 0.46 |  |

Large effect sizes (*A* ≤ 0.2 or *A* ≥ 0.8) are reported in bold

NAs indicate population extinction

Unperturbed baseline settings are listed in Table S1

# **TABLE S3**

Spearman Partial Rank Correlation Coefficients obtained from global sensitivity analysis of parameter perturbations using Latin-hypercube sampling. Coefficients represent the degree of association between response variables and model parameters while controlling for variation in other parameters.

|  | Sex ratio | | | Frequency of resistance alleles | | | Frequency of coercion alleles | | | |
| --- | --- | --- | --- | --- | --- | --- | --- | --- | --- | --- |
| Parameter | core | edge | difference | core | edge | difference | core | edge | difference |  |
| Probability of dispersal  ($\gamma$) | **0.33***** | **0.32**** | 0.06 | 0.05 | 0.04 | -0.03 | 0.04 | **0.20*** | -0.15 |  |
| Intensity of sexual conflict ($m$) | **0.85***** | **0.72***** | **0.38***** | **0.90***** | **0.88***** | **0.41***** | **0.94***** | **0.94***** | **0.45***** |  |
| Steepness of ecocline  () | -0.10 | **-0.21*** | **0.22*** | -0.02 | **-0.41***** | **0.45***** | -0.03 | **-0.27**** | **0.32**** |  |
| Relative fecundity of parthenogenesis ($\varepsilon$) | **0.89***** | **0.83***** | **0.28**** | **0.88***** | **0.78***** | **0.58***** | **0.90***** | **0.86***** | **0.60**** |  |

Positive and negative coefficients indicate that response variables increase and decrease, respectively, with increasing parameter values

Significant correlations are reported in bold

* denotes *p*<0.05; ** denotes *p*<0.01; *** denotes *p*<0.001

#
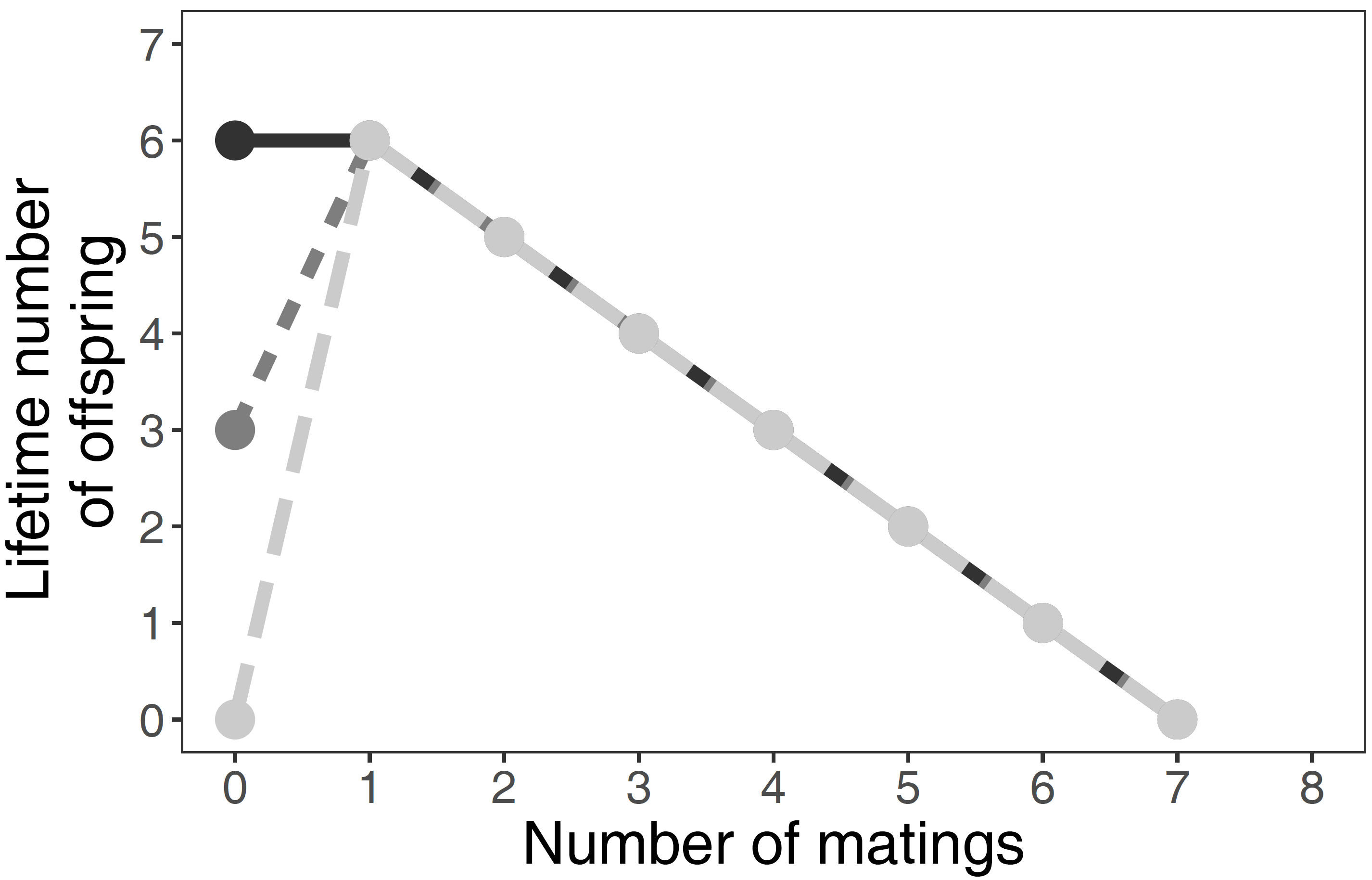


# **FIGURE S1**

Examples of the piecewise fitness functions described in equations 2a and 2b for obligately sexual (light grey) and mutant females (mid and dark grey) originating from the same habitat ($v$ = 1) with the same resistance-cost function ($\Phi$= 1). Other parameter settings for these functions are: $m$ = 1, $a$ = 7, $\varepsilon$ = 1 (dark grey) and $\varepsilon$ = 0.5 (mid grey).

#
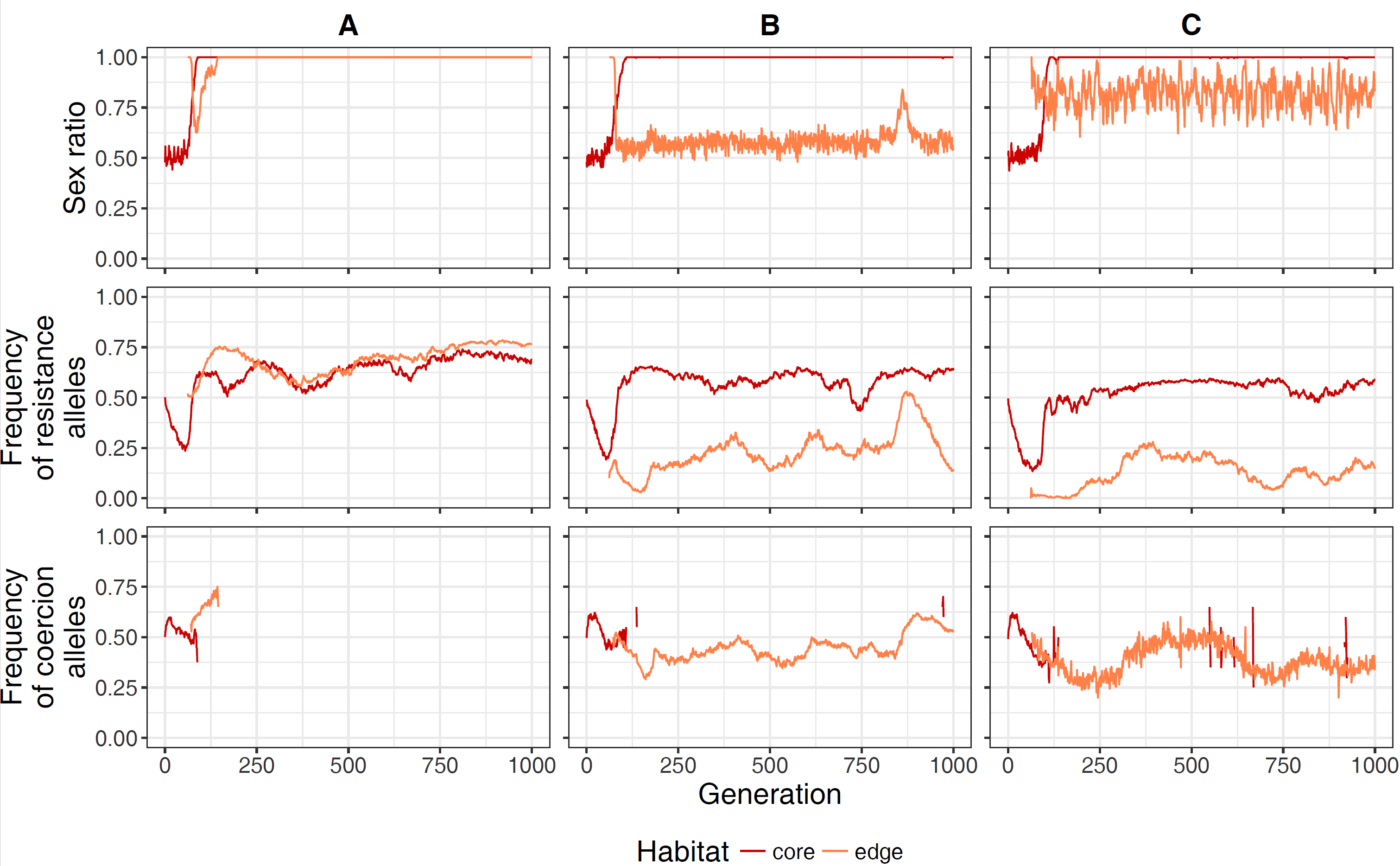
**FIGURE S2**

Time-series plots showing temporal patterns in distributions of sex ratio, frequency of pooled resistance alleles, and frequency of pooled coercion alleles between the core (red) and the edge (orange) assuming no ecocline ( = 0) [A], a shallow ecocline ( = 0.4) [B], and a steep ecocline ( = 0.7) [C]. Missing sections of line in coercion plots represent periods where males are locally extinct. Data are taken from single simulation runs lasting 1000 generations each. Other parameters: small refugia, $m$ = 1.5, $\varepsilon$ = 0.9.

#
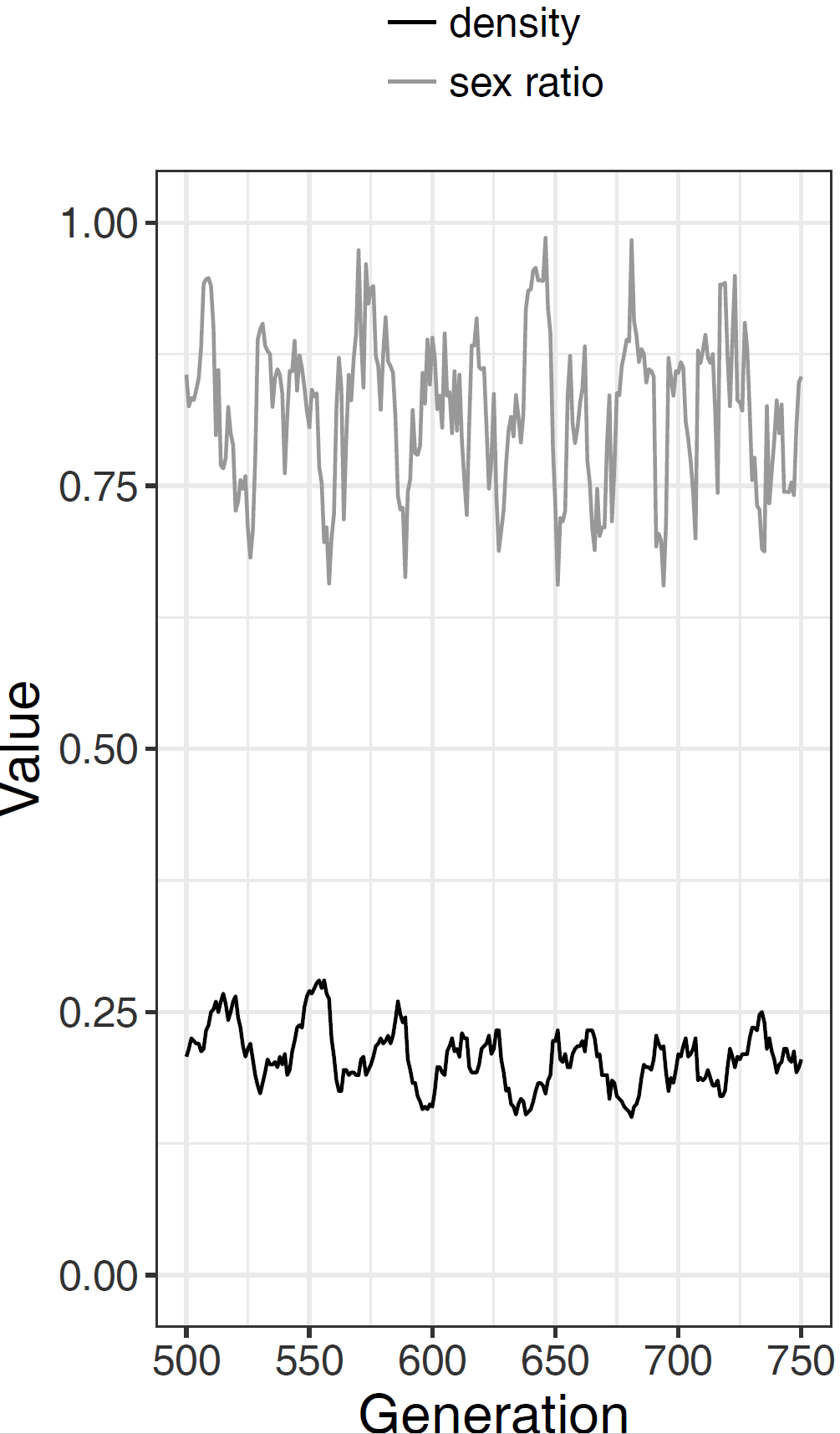


# **FIGURE S3**

Time-series snapshot of density and sex ratio at the edge under a steep ecocline (= 0.7). The high variance in sex ratio (grey line) is explained by frequency dependent selection: sex is favoured as density increases (resulting in dips in female bias), whereas parthenogenesis is favoured as density declines (resulting in peaks in female bias). Data are from a single simulation. Other settings: small refugia, $\varepsilon$ = 0.9, $m$ = 1.5.

#
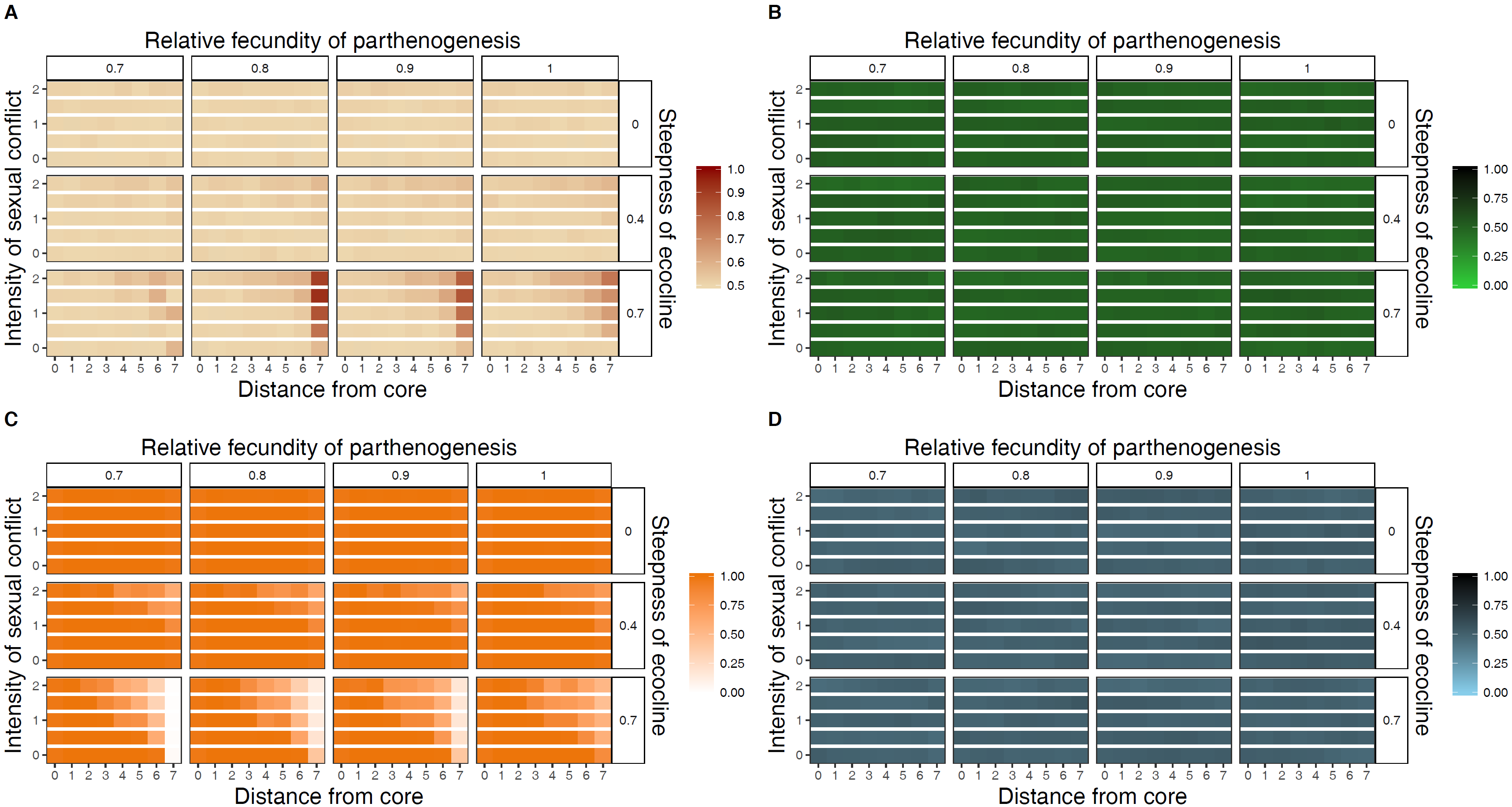


# **FIGURE S4**

Heat map showing spatial patterns for sex ratio (proportion female) [A], frequency of pooled resistance alleles [B], population density [C], and frequency of pooled coercion alleles [D] when resistance alleles are phenotypically neutral. Outcomes are median proportions obtained from 25 simulation runs lasting 500 generations each. Other parameter settings: no refugia.

#
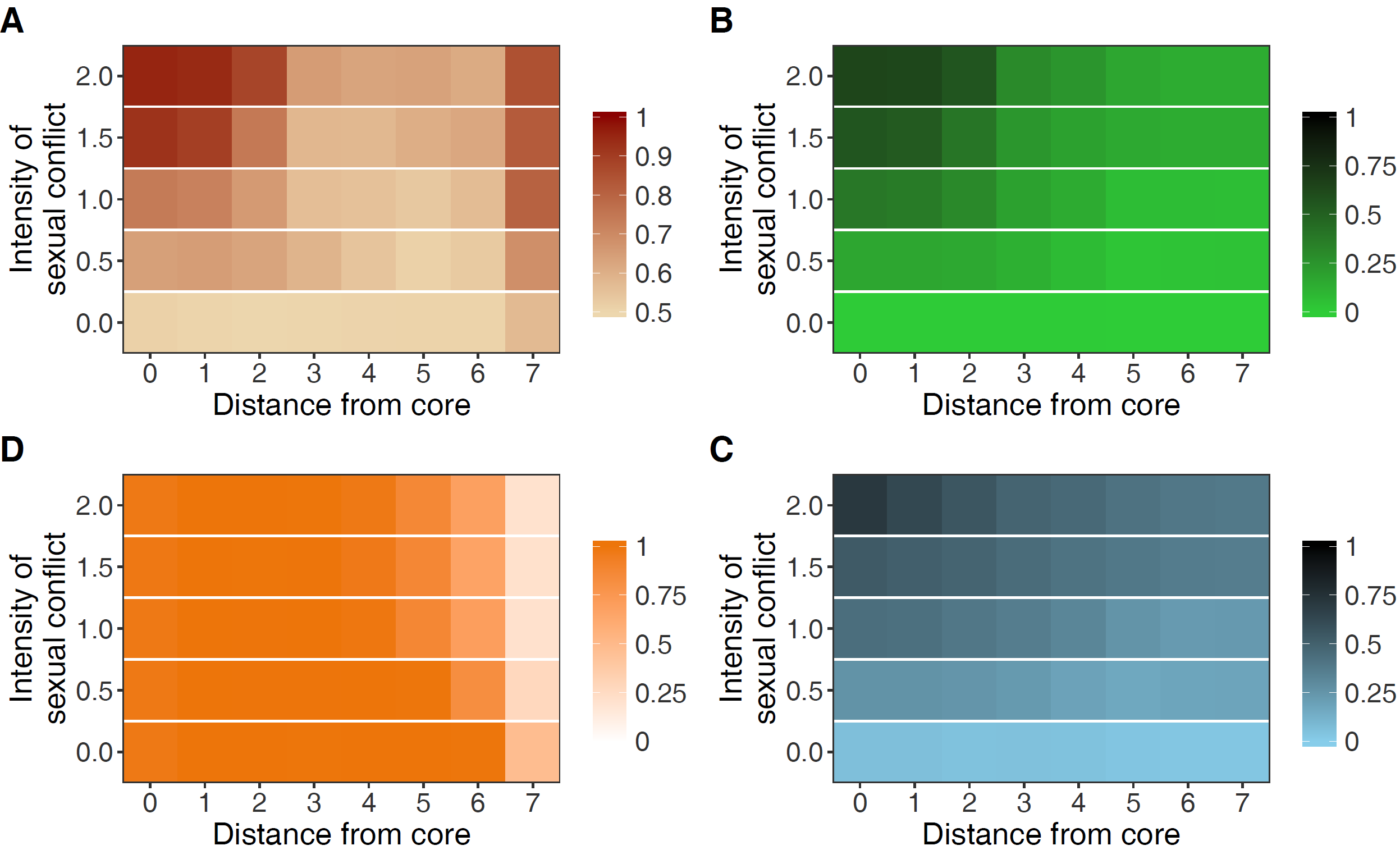
**FIGURE S5**

Heat map showing spatial patterns for sex ratio (proportion female) [A], frequency of pooled resistance alleles [B], population density [C], and frequency of pooled coercion alleles [D] under ongoing sexual coevolution following the introduction of the $P$ allele as a single allele mutation in 1% of individuals. Outcomes are median proportions obtained from 50 simulation runs lasting 500 generations each. Other settings: no refugia, $\varepsilon$ = 0.9, = 0.7.

#
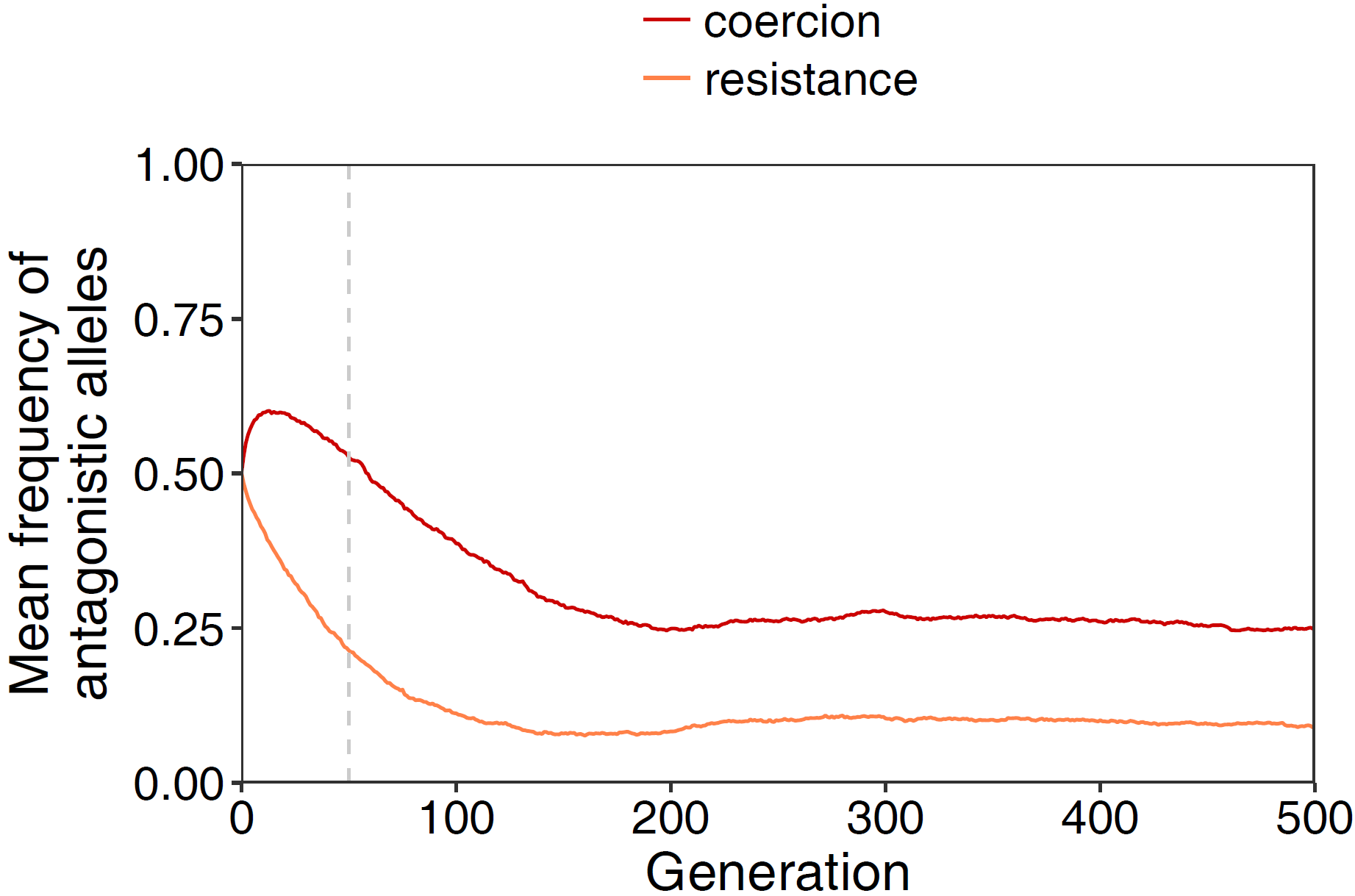


# **FIGURE S6**

Line graph showing the decline in average coercion and resistance allele frequencies of the sexual metapopulation over generational time for 25 simulation runs without introduction of the $P$ allele. The dotted line indicates the end of the burn-in phase and the time at which the $P$ allele is normally introduced into the model. Generation 50 was chosen as the time to introduce the P mutation as antagonistic allele frequencies where sharply declining at this point but standing genetic variance in coercion and resistance was still substantial in all simulation runs. Other settings: large refugia, $\varepsilon$ = 0.9, = 0.7, $m$ = 2.
